# Integration of family planning and nutrition programmes in 64 WHO member states of Africa, Eastern Mediterranean and South-East Asia regions: findings from a survey of Ministry of Health officials

**DOI:** 10.1136/bmjgh-2025-020307

**Published:** 2026-02-17

**Authors:** Iqbal H Shah, Moazzam Ali, Ndema A Habib, Ayaz Ahmed Baloch, Leopold Ouedraogo, Adeniyi Aderoba, Karima Gholbzouri, Meera T Upadhyay, Anoma Jarathilake, Wafaie Fawzi, Uttara Partap

**Affiliations:** 1 Department of Global Health and Population, Harvard T.H. Chan School of Public Health, Boston, Massachusetts, USA; 2 Department of Sexual and Reproductive Health and Research, World Health Organization, Geneve, Switzerland; 3 Faculty of Medicine and Health, The University of Sydney, Sydney, New South Wales, Australia; 4 World Health Organization Regional Office for Africa, Brazzaville, Republic of Congo; 5 World Health Organization Regional Office for the Eastern Mediterranean, Cairo, Egypt; 6 World Health Organization Regional Office for South-East Asia, New Delhi, India

**Keywords:** global health, nutrition, public health, cross-sectional survey

## Abstract

**Background:**

Family planning (FP) and nutrition services are essential for the health and well-being of girls and women in low- and middle-income countries (LMICs), yet evidence on their existing and potential integration is limited. Also, little is known about relevant national programmes and the views of health officials.

**Methods:**

An on-line survey of national health ministry officials in member states of WHO Regions for Africa (AFRO), Eastern Mediterranean (EMRO) and South-East Asia (SEARO) was conducted from 29 August 2023 to 15 December 2023, using a structured questionnaire. It covered national FP and nutrition programmes and policies, special FP programmes for vulnerable groups, task sharing in provision of services, and existing or potential for integration of FP and nutrition services. This was complemented with recent data from the UN Population Division and Global Health Observatory.

**Results:**

Altogether, 64 (81%) of 79 member states’ health officials responded. All countries in SEARO, 83% of countries in AFRO and 92% in EMRO reported the existence of a national nutritional programme. In 40 countries with integrated FP and nutrition programmes, integration of FP with breastfeeding counselling during the first 2 days after delivery was universal, followed by nutritional information, education and communication (98%) and nutritional counselling during pregnancy (98%). In 24 countries without an integrated FP and nutrition programme, food and/or cash assistance for nutrition and weekly iron folic acid supplement for women aged 15–49 years were reported as best suited for integration with FP. Integration with nutrition service was considered suitable by 58% of respondents.

**Conclusions:**

WHO regions vary widely in policies, programmes, provision and integration of FP and nutrition services. Officials of several countries recognise the potential values of integration, indicating the need to comprehensively explore strategies to promote integration to benefit women in LMICs.

**WHAT IS ALREADY KNOWN ON THIS TOPIC**

- Previously, a survey with a limited number of questions on family planning (FP) programmes was conducted in 2009 and 2015 among health ministry officials in countries of the WHO’s Eastern Mediterranean region.
- This previous survey indicated important gaps in the implementation of FP programmes and in addressing the needs of vulnerable groups but did not address the existence and alignment of policies and programmes in nutrition—another key area important to women’s health and well-being.

**WHAT THIS STUDY ADDS**

- We expanded the scope of the previous survey by adding additional topics on provision of FP and nutrition services including questions regarding task sharing, existing integration of FP and nutrition services and potential for integration of these services.
- We also expanded the coverage to include 52 countries in two additional WHO regions of Africa and South-East Asia.

**HOW THIS STUDY MIGHT AFFECT RESEARCH, PRACTICE OR POLICY**

- This study provides information on current coverage and gaps in the provision of FP and nutrition services and the potential for integration of the two.
- It also identifies the services reported as best suited for integration to better address the needs of women of reproductive age in low- and middle-income countries.

## Introduction

 Nutrition and family planning (FP) are vital for women’s survival and well-being in low- and middle-income countries (LMICs) and their service integration reinforces their benefits. However, these services are sometimes unavailable, provided through vertical programmes or are out of reach for groups most in need. Integrating FP with other services has been a long-standing priority in LMICs.^1^ Integration of FP and nutrition was highlighted for mutually reinforcing impact in addressing malnutrition and high fertility as far back as in 1974 when these programmes were being established in LMICs.^2^ Integrating healthcare and related services, including FP and nutrition, continues to be highlighted as a key strategy to increase impact for women’s health and improve their access to effective care.^3^,^5^ Furthermore, evidence continues to accumulate on the epidemiological and biological relationship between the two. FP can influence women’s nutritional status through preventing adolescent pregnancy, increasing birth spacing and enabling smaller families, and hormonal contraception may also exert positive effects on haemoglobin, potentially through reduced blood loss from menstruation.^6^,^11^

Most studies show positive impact on health outcomes when FP is integrated with delivery of maternity services,^12^ infant and child immunisation^13^ and services for HIV/sexually transmitted infections.^14^ However, a systematic review concluded that the evidence on the integration of nutrition with FP, adolescent health and reproductive health services was limited.^15^ Few studies have therefore examined the potential and impact of integrating FP with nutrition services.^16^ This is despite the multiple potential contact points that present opportunities during which FP and nutrition services could be co-delivered throughout the life course, including during adolescence, during adulthood in the prepregnancy period, antenatally, at delivery and post partum.^16^

Even fewer studies have ascertained the information from and views of national health officials on this topic. A recent study sought perspectives of a range of stakeholders on integrating FP and nutrition services.^17^ Overall, stakeholders noted that integration is potentially beneficial for increasing access, improving outcomes and increasing cost-effectiveness by using the same service platforms and staff. This study was a qualitative study of global stakeholders, which included only a few health officials from LMICs who make and implement health programmes.^17^ Obtaining information and views from national health officials in this regard is essential to gaining a clearer picture of the current landscape of FP and nutrition service integration across countries, as well as gauging the extent to which this integration is considered as a topic of importance at the national level. Such evidence would be key to shaping approaches that most suitably advance integration or alignment of FP and nutrition services in a manner that is accepted and supported by governments and best suited to national contexts.

With the aim to map national policies and programmes on FP and nutrition, we conducted a survey exploring information and views from health officials of member states of WHO regions for Africa (AFRO), Eastern Mediterranean (EMRO) and South-East Asia (SEARO). The specific objectives of the survey were to: (1) map national policies and programmes in FP and nutrition; (2) understand the specific FP and nutrition services available across health service levels and for specific population groups; (3) ascertain actual integration and potential opportunities for integration of FP with nutrition services and (4) examine task sharing in the provision of FP and nutrition information, counselling and services.

The current descriptive study focuses on information obtained from this survey regarding the integration of FP with nutrition in member states of the WHO regional offices of AFRO, EMRO and SEARO.

## Methods

Building on previous experiences of cross-sectional surveys of Ministry of Health officials of EMRO member states undertaken by WHO that specifically examined policies and programmes for FP,^18^ a similar methodology was used in the current study with expanded scope and coverage. Using the survey tool that was successfully implemented and accepted for regional assessment in 2009 and 2015 among member states of EMRO as the starting point,^18 19^ additional questions were developed in consultation with the relevant staff of WHO regional offices and technical experts in FP and nutrition research. Topics covered in the tool were on: national policies and programmes in FP and nutrition; availability of type of contraceptives at the outreach, primary healthcare (PHC) and hospital level; existence of special FP programmes for vulnerable groups; currently existing and potential opportunities for integration of FP with other sexual and reproductive health (SRH) and nutritional services and task sharing in the provision of FP and nutrition information, counselling and methods/supplements by cadres of health providers. ‘Integration’ was defined in the tool as the delivery of multiple programmes or services across the same points of contact, delivered by the same provider or delivered on the same service platform. The tool was pretested with selected health officials of countries in three regions. We reviewed pretest responses for consistency, skip patterns and phrasing of questions. The questionnaire was revised considering the experience from the pretest. It was implemented through the survey platform SurveyMonkey (https://www.surveymonkey.com/), with the WHO regional offices taking responsibility for data collection from country officials of Ministries of Health (reflexivity statement). The survey protocol called for a maximum of three reminders via email from the regional focal point person to the country offices of WHO in direct contact with local health officials identified as the ‘respondent’ for the survey.

### Patient and public involvement

No patients or members of the public were involved in this research.

### Eligible respondents

The respondents eligible to complete the online questionnaire were specified as National Programme Officer for Population and/or Family Planning and/or for Maternal, Newborn and Child Health (MNCH) at the National Ministry of Health, and were identified by the participating country offices of WHO that recruited one respondent per country. Respondents were instructed to seek input from relevant officials for questions that fell outside their domain to complete all questions. In cases where the identified respondent considered that another official within the Ministry was better placed to address specific questions, they were requested to coordinate or delegate the response accordingly. Most respondents completed questionnaires on their own. The survey protocol also set the minimum target of 70% for the response rate for all countries combined as a threshold for coverage to proceed to analysis. This figure was chosen based on previous studies discussing standards in survey research, where a response rate of 70% or higher has been considered acceptable to minimise non-response bias.^20 21^

### Data collection and analysis

Following the identification and acceptance of the survey respondents, the survey link was sent to them. The survey tool was self-administered online using SurveyMonkey and the data were archived and checked for quality in terms of consistency and completeness.

The survey was designed to map information on policies and document perspectives of health officials in all countries in each region. Therefore, we did not design a sampling strategy or calculate a target sample size. Given the sample size of 64 and confidence level of 95%, the margin of error for a prevalence of 62% of integration of FP and nutrition programmes was retrospectively calculated to be ±11.9%.

We used simple descriptive measures of frequencies and cross-tabulation for analysis of data. All analysis was conducted using STATA V.14 (StataCorp, College Station, Texas, USA). All analyses were conducted overall for countries in three regions combined and stratified by WHO region. We first examined the number and percentage of countries reporting the existence of national nutrition and FP policies and programmes, including the characteristics of such programmes. We then examined the number and percentage of countries reporting any integration of FP and nutrition services, overall and by components of those services, and further explored which services were typically integrated and which offered the option of integration. We also examined the current level of overlap in service delivery for FP and nutrition among different cadres of health service providers and among existing social protection platforms. Where relevant, for percentages, we generated 95% confidence intervals calculated using the Wilson method. No survey weights or clustering adjustments were applied.

To provide additional descriptive context, we complemented survey data with available national-level data on FP and nutrition indicators that were available from the UN Population Division (https://population.un.org/wpp/Download/Standard/Population/ and https://www.un.org/development/desa/pd/data/world-contraceptive-use), and the WHO Global Health Observatory (https://www.who.int/data/gho/data/indicators).

In sensitivity analyses, we examined country-level differences in key FP and nutrition indicators by responding versus non-responding countries, and checked for differential responses by the respondent background (FP; maternal, newborn and child health; sexual and reproductive health and public health or other), focusing specifically on responses relating to potential for integration.

## Results

Data collection started on 29 August and ended on 15 December 2023. Responses from 64 of the 79 member states of three regions were received, indicating a response rate of 81%. The 64 respondents included National Programme Officer for: FP (n=19); maternal, newborn and child health (n=17); sexual and reproductive health (n=11); public health or other (n=17) at the national Ministries of Health of member states. The response rate of 91% was the highest for SEARO with all (n=11) member states, except one, responding to the survey. With 42 of 47 member states of AFRO providing information, the response rate was 89% while EMRO had the lowest response rate of 57% with 12 of its 21 member states providing information. The population of 64 countries comprised 45% of the world population in 2022.^22^

We primarily focus on results related to the integration of FP with nutrition services by first describing the context, followed by different aspects of integration.

### The context of family planning and nutrition: prevalence, programmes, policies and provision of services

The countries surveyed ranged widely on most indicators (online supplemental table A.1). The prevalence of all contraceptive methods was the lowest (0.9%) in Somalia and the highest in Thailand (71.6%). The prevalence of modern contraceptive methods (mCPR) was <15% in 13, 15%–29% in 22, 30%–44% in 10 and 45% or higher in 19 countries. Unmet need for FP was the lowest (8%) in Sri Lanka and Thailand and the highest in Libya (40.2%). Diversity in these indicators was more visible in countries of AFRO than of EMRO or SEARO. The proportion of non-pregnant women aged 15–49 years with anaemia ranged from 17.2% in Rwanda to over 50% in Gabon, Benin, Burkina Faso, Côte d’Ivoire, Mali, Nigeria and Senegal. When considering pregnant women, the number of countries with over 50% prevalence of anaemia was higher (n=11) (online supplemental table A.1).

We examined if countries whose officials responded to the survey differed from those whose officials did not, on selected FP and nutrition indicators. Online supplemental table A.2 shows that there were no significant differences between such countries in mCPR or unmet need for FP, overall and by region. On the other hand, the prevalence of anaemia was higher among responding countries overall and in EMRO versus non-responders.

### Characteristics of family planning and nutrition programmes in survey countries

The provision of FP information, education and communication (IEC) was reported widespread at all three levels of healthcare—outreach (including community, home, schools, subcentres, mobile clinics and satellite clinics), primary healthcare (PHC) and hospital (table 1). The provision of FP IEC at PHC level was reportedly universal in both SEARO and EMRO as compared with 95.2% in AFRO countries. The provision of IEC at outreach level was reported the least in EMRO (58.3%) compared with AFRO (95.2%) and SEARO (100%). In the 64 countries, the provision of FP prescriptions/referrals and modern spacing methods was overall more often reported at the PHC level (78.1% and 92.2%, respectively) than at outreach (51.6% and 64.1%, respectively) or at the hospital level (67.2% and 90.6%, respectively). As expected, the provision of male or female sterilisation was primarily at the hospital level (92.2%).

**Table 1:** Number and percentage (%) of countries by reported characteristics of national family planning programmes, by region

Indicator	AFRO N=42 n, % (95% CI)	SEARO N=10 n, % (95% CI)	EMRO N=12 n, % (95% CI)	All regions N=64 n, % (95% CI)
Provision of IEC at:				
(a) Outreach level	40, 95.2 (84.2 to 98.7)	10, 100.0 (72.2 to 100.0)	7, 58.3 (32.0 to 80.7)	57, 89.1 (79.1 to 94.6)
(b) PHC	40, 95.2 (84.2 to 98.7)	10, 100.0 (72.2 to 100.0)	12, 100.0 (75.8 to 100.0)	62, 96.9 (89.3 to 99.1)
(c) Hospital	39, 92.9 (81.0 to 97.5)	10, 100.0 (72.2 to 100.0)	9, 75.0 (46.8 to 91.1)	58, 90.6 (81.0 to 95.6)
Provision of prescriptions/referrals at:				
(a) Outreach level	23, 54.8 (39.9 to 68.8)	6, 60.0 (31.3 to 83.2)	4, 33.3 (13.8 to 60.9)	33, 51.6 (39.6 to 63.4)
(b) PHC	32, 76.2 (61.5 to 86.5)	9, 90.0 (59.6 to 98.2)	9, 75.0 (46.8 to 91.1)	50, 78.1 (66.6 to 86.5)
(c) Hospital	28, 66.7 (51.6 to 79.0)	7, 70.0 (39.7 to 89.2)	8, 66.7 (39.1 to 86.2)	43, 67.2 (55.0 to 77.4)
Provision of modern spacing methods at:				
(a) Outreach level	32, 76.2 (61.5 to 86.5)	7, 70.0 (39.7 to 89.2)	2, 16.7 (4.7 to 44.8)	41, 64.1 (51.8 to 74.7)
(b) PHC	39, 92.9 (81.0 to 97.5)	10, 100.0 (72.2 to 100.0)	10, 83.3 (55.2 to 95.3)	59, 92.2 (83.0 to 96.6)
(c) Hospital	40, 95.2 (84.2 to 98.7)	10, 100.0 (72.2 to 100.0)	8, 66.7 (39.1 to 86.2)	58, 90.6 (81.0 to 95.6)
Provision of male/female sterilisation at:				
(a) Outreach level	6, 14.3 (6.7 to 27.8)	1, 10.0 (1.8 to 40.4)	0, 0.0 (0.0 to 24.2)	7, 10.9 (5.4 to 20.9)
(b) PHC	6, 14.3 (6.7 to 27.8)	3, 30.0 (10.8 to 60.3)	1, 8.3 (1.5 to 35.4)	10, 15.6 (8.7 to 26.4)
(c) Hospital	40, 95.2 (84.2 to 98.7)	10, 100.0 (72.2 to 100.0)	9, 75.0 (46.8 to 91.1)	59, 92.2 (83.0 to 96.6)
National FP guidelines regularly updated	41, 97.6 (87.7 to 99.6)	9, 90.0 (59.6 to 98.2)	10, 83.3 (55.2 to 95.3)	60, 93.8 (85.0 to 97.5)
FP services incorporated into social protection programmes	26, 61.9 (46.8 to 75.0)	8, 80.0 (49.0 to 94.3)	6, 50.0 (25.4 to 74.6)	40, 62.5 (50.3 to 73.3)
Special FP programmes exist for adolescents aged 10–14 years	30, 71.4 (56.4 to 82.8)	8, 80.0 (49.0 to 94.3)	3, 25.0 (8.9 to 53.2)	41, 64.1 (51.8 to 74.7)
Special FP programmes exist for adolescents aged 15–19 years	38, 90.5 (77.9 to 96.2)	9, 90.0 (59.6 to 98.2)	5, 41.7 (19.3 to 68.0)	52, 81.3 (70.0 to 88.9)

Overall percentages across all 64 countries are not weighted by the number of countries in each region.

AFRO, African Region; CI, confidence interval; EMRO, Eastern Mediterranean Region; FP, family planning; IEC, information, education and communication; PHC, primary healthcare; SEARO, South-East Asia Region.

The regional differences in the reported provision of FP services were marked, with EMRO countries lagging AFRO and SEARO for any type of FP service at all levels of healthcare. Countries in SEARO, on the other hand, reported the highest levels of provision, except for modern spacing methods at the outreach level (table 1).

National guidelines for FP were reported for most countries and were reported to be regularly updated by 97.6% of AFRO, 90% of SEARO and 83.3% of EMRO countries. Nearly two-thirds of all 64 countries reported incorporation of FP services into social protection programmes for vulnerable groups with the highest proportion (80%) in SEARO, followed by AFRO (61.9%) and EMRO (50%) (table 1).

The survey enquired about the existence of programmes for vulnerable groups to address their special needs. Overall, 64.1% of 64 countries reported special FP programmes for adolescents aged 10–14 years and 81.3% for adolescents aged 15–19 years. Fewer countries in EMRO reported existence of special FP programmes for young adolescents aged 10–14 years (25%) compared with AFRO (71.4%) or SEARO (89%). This pattern continued for adolescents aged 15–19 years but with an increasing number of EMRO countries (41.7%) with special programmes in FP. This compares with 90.5% of countries in AFRO and 90% of countries in SEARO (table 1).

Table 2 shows the reported characteristics of national nutrition programmes. Fifty-six of 64 countries (87.5%) reported the existence of a national nutrition programme and 33 (51.6%) of these 64 provided nutrition services as a stand-alone service. All countries in SEARO had a national nutrition programme as compared with 91.7% of countries in EMRO and 83.3% in AFRO. Stand-alone nutritional services were reported by 60%, 41.7% and 52.4% of SEARO, EMRO and AFRO countries, respectively. More countries in AFRO and SEARO reportedly incorporated nutrition in social protection programmes for vulnerable groups than in EMRO. Overall, 76.6% of 64 countries reported incorporating nutrition into social protection programmes. This is compared with 52.5% for FP services reported in table 1. The highest proportion of countries with nutritional services in social protection programmes was in AFRO (81%).

**Table 2:** Number and percentage (%) of countries by reported characteristics of national nutrition programmes, by region

Indicator	AFRO N=42 n, % (95% CI)	SEARO N=10 n, % (95% CI)	EMRO N=12 n, % (95% CI)	All regions N=64 n, % (95% CI)
National nutrition programme in existence	35, 83.3 (69.4 to 91.7)	10, 100.0 (72.2 to 100.0)	11, 91.7 (64.6 to 98.5)	56, 87.5 (77.2 to 93.5)
Nutrition services provided as stand-alone service	22, 52.4 (37.7 to 66.6)	6, 60.0 (31.3 to 83.2)	5, 41.7 (19.3 to 68.0)	33, 51.6 (39.6 to 63.4)
Nutritional services incorporated into social protection programmes	34, 81.0 (66.7 to 90.0)	7, 70.0 (39.7 to 89.2)	8, 66.7 (39.1 to 86.2)	49, 76.6 (64.9 to 85.3)
Nutritional services delivered at:				
(a) Schools	30, 71.4 (56.4 to 82.8)	10, 100.0 (72.2 to 100.0)	7, 58.3 (32.0 to 80.7)	47, 73.4 (61.5 to 82.7)
(b) Home	26, 61.9 (46.8 to 75.0)	9, 90.0 (59.6 to 98.2)	6, 50.0 (25.4 to 74.6)	41, 64.1 (51.8 to 74.7)
(c) Clinic	38, 90.5 (77.9 to 96.2)	10, 100.0 (72.2 to 100.0)	9, 75.0 (46.8 to 91.1)	57, 89.1 (79.1 to 94.6)
Reported target groups for nutrition services and interventions				
(a) Infant and children (0–5 years)	40, 95.2 (84.2 to 98.7)	10, 100.0 (72.2 to 100.0)	9, 75.0 (46.8 to 91.1)	59, 92.2 (83.0 to 96.6)
(b) Adolescents (10–14 years)	32, 76.2 (61.5 to 86.5)	10, 100.0 (72.2 to 100.0)	5, 41.7 (19.3 to 68.0)	47, 73.4 (61.5 to 82.7)
(c) Adolescents (15–19 years)	25, 59.5 (44.5 to 73.0)	9, 90.0 (59.6 to 98.2)	6, 50.0 (25.4 to 74.6)	40, 62.5 (50.3 to 73.3)
(d) Women in preconception	20, 47.6 (33.4 to 62.3)	10, 100.0 (72.2, 100.0)	5, 41.7 (19.3 to 68.0)	35, 54.7 (42.6 to 66.3)
(e) Pregnant women	38, 90.5 (77.9 to 96.2)	10, 100.0 (72.2 to 100.0)	10, 83.3 (55.2 to 95.3)	58, 90.6 (81.0 to 95.6)
(f) Postpartum women	35, 83.3 (69.4 to 91.7)	10, 100.0 (72.2 to 100.0)	10, 83.3 (55.2 to 95.3)	55, 85.9 (75.4 to 92.4)

Overall percentages across all 64 countries are not weighted by the number of countries in each region.

AFRO, African Region; CI, confidence interval; EMRO, Eastern Mediterranean Region; FP, family planning; SEARO, South-East Asia Region.

The nutritional services were reportedly delivered mostly at clinics (89.1%), followed by schools (73.4%) and homes (73.4%). At each platform, SEARO had the highest reported provision (100% at schools and clinics and 90% at home) and EMRO the lowest (50% at home, 58.3% at schools and 75% at clinics) (table 2).

National nutritional programmes indicate groups that are targets for nutritional services and interventions. Overall, infants and children (0–59 months) were reported as targets for nutritional services in 92.2% of 64 countries, followed by pregnant women (90.6%) and postpartum women (85.9%). Women in preconception (ie, those not currently pregnant, but planning pregnancy in the foreseeable future^23^) were least often the target for nutritional interventions (54.7%). Five of six groups were identified by all countries of SEARO as targets for nutritional intervention. Adolescents aged 15–19 years were reported as the target group for nutrition services by nine of 10 SEARO countries. Adolescents aged 15–19 years and women in preconception were the least frequently reported target groups in AFRO (59.5% and 47.6%, respectively). In EMRO, the least frequently mentioned target groups were adolescents aged 10–14 years and women in preconception (41.7% for each) (table 2).

To overcome the shortage of health workers, especially those who are highly skilled, WHO recommends task sharing to optimise the provision of health services.^24^ WHO’s guidelines recommend that all cadres of health workers are capable of providing IEC and counselling on family planning and nutrition (FPN). Online supplemental table A.3 indicates the type of nutrition services reportedly provided by various healthcare cadres in 64 countries of the three regions. Overall, IEC on nutrition in 64 survey countries was provided mainly by nurses (90.6%), midwives (89.1%), non-specialist doctors (87.5%) and specialist doctors (89.1%) as well as by lay health workers (78.1%), such as community health workers (CHWs). Compared with the provision of IEC on FP (online supplemental figure A.1), more countries reported provision of IEC on nutrition by CHWs and auxiliary nurses than on FP. On the other hand, more countries reported that pharmacy workers and pharmacists provide IEC on FP than on nutrition. The reported provision of FP and nutrition IEC services by other cadres was similar. While clearly within the remit of such mid-level healthcare providers as auxiliary nurses and auxiliary nurse-midwives, very few of them were reported to provide IEC either for FP or nutrition. Provision of IEC on nutrition by any cadres of healthcare was the least common in countries of EMRO than of AFRO and SEARO. Nutrition counselling during pregnancy showed a similar pattern. Counselling during pregnancy on multiple-micronutrients and breastfeeding was provided mostly by the same cadres as other services—nurses, midwives, non-specialist and specialist doctors—while few countries reported pharmacy workers, pharmacists, auxiliary nurses and auxiliary nurse-midwives providing this service. Lay health workers notably provided counselling during pregnancy on breastfeeding (online supplemental table A.3).

WHO recommendations indicate that all cadres, except unsupervised lay health workers, can provide iron-folic acid (IFA) supplement to pregnant women,^24 25^ yet in <50% of 64 countries were auxiliary nurses, pharmacists and pharmacy workers reported to provide it. Nurses, midwives, non-specialist doctors and specialist doctors provided IFA supplements in over 80% of countries. No country of EMRO reported provision of IFA by lay health workers (CHWs) compared with 59.2% in AFRO and 50% in SEARO (online supplemental table A.3).

In comparison with IFA supplements to pregnant women, fewer countries indicated provision of weekly IFA supplement by any cadre to adolescents and reproductive-aged (15–49 years) women. Similarly, the provision of food and/or cash assistance to vulnerable population groups by any cadre was low and ranged from 7.8% by pharmacy workers to 28.1% by nurses and lay health workers (online supplemental table A.3).

The provision of vitamin A supplementation among children aged 6–59 months was largely by nurses, midwives, non-specialist and specialist doctors. Overall, the provision of various types of nutrition services was primarily by nurses, midwives, non-specialist and specialist doctors in a large number of 64 countries. The provision of such services by any cadre was less common in countries of EMRO than those of AFRO or SEARO (online supplemental table A.3).

### Integration of family planning with nutrition and other programmes

‘Integration’ was defined in the survey as delivery of multiple programmes across the same points of contact, delivered by the same provider or delivered on the same service platform. Of the 64 countries, 32 (50%) indicated provision of FP and nutrition services at the same delivery point or by the same provider while eight countries (12.5%) reported provision at the same point of contact. The proportion was the highest in SEARO, where 80% of countries co-delivered FP and nutrition at the same delivery point and 90% by the same provider. In SEARO, 80% of countries also indicated that FP and nutrition services are provided both as standalone and jointly (data not shown). Altogether, 40 (62.5%) of 64 countries reported FP counselling and 31 (48.4%) reported FP method provision currently integrated with nutrition services (table 3).

**Table 3:** Number and percentage (%) of countries indicating family planning currently integrated with nutrition services, by type of service and region

FP and nutrition integration	AFRO N=42 n, % (95% CI)	SEARO N=10 n, % (95% CI)	EMRO N=12 n, % (95% CI)	All regions N=64 n, % (95% CI)
FP counselling currently integrated with nutrition services	29, 69.0 (54.0 to 80.9)	8, 80.0 (49.0 to 94.3)	3, 25.0 (8.9 to 53.2)	40, 62.5 (50.3 to 73.3)
FP method provision currently integrated with nutrition services	21, 50.0 (35.5 to 64.5)	7, 70.0 (39.7 to 89.2)	3, 25.0 (8.9 to 53.2)	31, 48.4 (36.6 to 60.4)
Specific nutritional service integrated with FP in 40 countries with FPN integrated programmes*	**N=29**	**N=8**	**N=3**	**N=40**
Nutritional information, education and communication only	29, 100.0 (88.3 to 100.0)	8, 100.0 (67.6 to 100.0)	2, 66.7 (20.8 to 93.9)	39, 97.5 (87.1 to 99.6)
Vitamin A supplementation among children aged 6–59 months	20, 69.0 (50.8 to 82.7)	5, 62.5 (30.6 to 86.3)	0, 0.0 (0.0 to 56.1)	25, 62.5 (47.0 to 75.8)
Weekly IFA supplement for women aged 15–49 years	12, 41.4 (25.5 to 59.3)	6, 75.0 (40.9 to 92.9)	2, 66.7 (20.8 to 93.9)	20, 50.0 (35.2 to 64.8)
Daily IFA supplement for pregnant women	23, 79.3 (61.6 to 90.2)	7, 87.5 (52.9 to 97.8)	2, 66.7 (20.8 to 93.9)	32, 80.0 (65.2 to 89.5)
Multiple micronutrient supplement for pregnant women	16, 55.2 (37.5 to 71.6)	5, 62.5 (30.6 to 86.3)	3, 100.0 (43.9 to 100.0)	24, 60.0 (44.6 to 73.7)
Food and/or cash assistance to population groups	13, 44.8 (28.4 to 62.5)	7, 87.5 (52.9 to 97.8)	1, 33.3 (6.1 to 79.2)	21, 52.5 (37.5 to 67.1)
Nutrition counselling during pregnancy	29, 100.0 (88.3 to 100.0)	8, 100.0 (67.6 to 100.0)	2, 66.7 (20.8 to 93.9)	39, 97.5 (87.1 to 99.6)
Breastfeeding counselling during pregnancy	28, 96.6 (82.8 to 99.4)	8, 100.0 (67.6 to 100.0)	2, 66.7 (20.8 to 93.9)	40, 95.0 (83.5 to 98.6)
Breastfeeding counselling during the first 2 days after delivery	29, 100.0 (88.3 to 100.0)	8, 100.0 (67.6 to 100.0)	3, 100.0 (43.9 to 100.0)	40, 100.0 (91.2 to 100.0)

Overall percentages across all 64 countries are not weighted by the number of countries in each region.

* Countries that reported integration of FP counselling or method provision with nutrition are the sample (denominator) for the analysis.

AFRO, African Region; CI, confidence interval; EMRO, Eastern Mediterranean Region; FP, family planning; FPN, family planning and nutrition; IFA, iron-folic acid; SEARO, South-East Asia Region.

### When family planning and nutrition programmes are integrated: differentials in selected indicators

For 40 countries that reported currently integrated FPN programmes, we examined the type of nutrition service integrated with FP (table 3). The provision of IEC on nutrition was the most reported currently integrated service with FP in 39 of the 40 countries with FPN integrated programmes. IEC on nutrition was reportedly integrated with FP by all countries of AFRO and SEARO. Nutrition counselling during pregnancy and counselling on breastfeeding during the first 2 days after delivery were universally reported as integrated with FP information and counselling in all countries in AFRO and SEARO. On the other hand, the provision of weekly IFA supplement for women aged 15–49 years and food and/or cash assistance to vulnerable population groups was less integrated with FP programmes (table 3). There appeared to be no significant differences in responses identifying integrated services by the domain of the respondent (online supplemental table A.4).

### Potential for integrating specific nutrition services with family planning

For 24 countries that reported currently non-existent integrated FPN programmes, we examined the potential for integration of specific types of nutrition services (table 4). There were only two countries (Sri Lanka and Bhutan) in SEARO that reported no FPN integrated programme. In countries reporting no existing integrated FPN programmes, the interest in integrating specific nutrition service with FP was modest, except for food and/or cash assistance (54%) and weekly IFA supplement for women aged 15–49 years (46%). Countries in SEARO and EMRO reported low interest in integration of any nutrition service with FP. On the other hand, two-thirds or more countries in AFRO expressed interest in integration of food and/or cash assistance to population groups, nutrition counselling during pregnancy and breastfeeding counselling during pregnancy with FP. Responses about potential for FP and nutrition service integration were not significantly different by the domain of the respondent (online supplemental table A.5).

**Table 4:** Number and percentage (%) of countries with currently no integrated FPN programmes indicating positively the specific nutritional service for integration with FP services, by type of service and region

FP and nutrition integration	AFRO N=13 n, % (95% CI)	SEARO N=2 n, % (95% CI)	EMRO N=9 n, % (95% CI)	All regions N=24 n, % (95% CI)
Specific nutritional service for integration with FP in 24 countries with no FPN integrated programmes*				
Nutritional information, education and communication only	5, 38.5 (17.7 to 64.5)	0, 0.0 (0.0 to 65.8)	1, 11.1 (2.0 to 43.5)	6, 25.0 (12.0 to 44.9)
Vitamin A supplementation among children aged 6–59 months	7, 53.8 (29.1 to 76.8)	0, 0.0 (0.0 to 65.8)	2, 22.2 (6.3 to 54.7)	9, 37.5 (21.2 to 57.3)
Weekly IFA supplement for women aged 15–49 years	8, 61.5 (35.5 to 82.3)	0, 0.0 (0.0 to 65.8)	3, 33.3 (12.1 to 64.6)	11, 45.8 (27.9 to 64.9)
Daily IFA supplement for pregnant women	7, 53.8 (29.1 to 76.8)	0, 0.0 (0.0 to 65.8)	1, 11.1 (2.0 to 43.5)	8, 33.3 (18.0 to 53.3)
Multiple micronutrient supplement for pregnant women	7, 53.8 (29.1 to 76.8)	0, 0.0 (0.0 to 65.8)	2, 22.2 (6.3 to 54.7)	9, 37.5 (21.2 to 57.3)
Food and/or cash assistance to population groups	10, 76.9 (49.7 to 91.8)	0, 0.0 (0.0 to 65.8)	3, 33.3 (12.1 to 64.6)	13, 54.2 (35.1 to 72.1)
Nutrition counselling during pregnancy	9, 69.2 (42.4 to 87.3)	0, 0.0 (0.0 to 65.8)	0, 0.0 (0.0 to 29.9)	9, 37.5 (21.2 to 57.3)
Breastfeeding counselling during pregnancy	9, 69.2 (42.4 to 87.3)	0, 0.0 (0.0 to 65.8)	0, 0.0 (0.0 to 29.9)	9, 37.5 (21.2 to 57.3)
Breastfeeding counselling during the first 2 days after delivery	8, 61.5 (35.5 to 82.3)	0, 0.0 (0.0 to 65.8)	1, 11.1 (2.0 to 43.5)	9, 37.5 (21.2 to 57.3)

Respondents were provided with a predefined list of services (as above) and asked which they considered suitable for integration with FP services.

Overall percentages across all 64 countries are not weighted by the number of countries in each region.

* Countries that reported *no* integration of FP counselling *or* method provision with nutrition are the sample (denominator) for the analysis.

AFRO, African Region; CI, confidence interval; EMRO, Eastern Mediterranean Region; FP, family planning; FPN, family planning and nutrition; IFA, iron-folic acid; SEARO, South-East Asia Region.

Regarding the potential of integrating FP and nutrition programmes, we considered overlap in delivery of these services by the same healthcare cadre and service delivery platform (table 5). Co-delivery of some aspects of FP and nutrition services by each type of healthcare cadre was much more common than provision of each one alone or none of the two. For example, 78.1% of lay health workers such as CHWs in all countries reported providing some aspects of FP and nutrition services. Nurses, midwives, non-specialist doctors and specialist doctors in over 90% of countries provide services for both FP and nutrition. This high degree of co-delivery of FP and nutrition services, particularly by lay health workers at the community level and by healthcare providers at the clinical level, suggests high potential for ensuring aligned services at these levels. Similarly, social protection programmes for vulnerable groups in over 50% of countries were reported to cater for both FP and nutrition services.

**Table 5:** Overlap between service delivery cadres and platforms for FP and nutrition, across all 64 surveyed countries (n, %, 95% CI)

	Neither FP nor nutrition	FP only	Nutrition only	FP and nutrition
Cadre of health worker				
(a) Lay health worker (eg, CHW)	7, 10.9 (5.4 to 20.9)	4, 6.3 (2.5 to 15.0)	3, 4.7 (1.6 to 12.9)	50, 78.1 (66.6 to 86.5)
(b) Pharmacy worker	14, 21.9 (13.5 to 33.4)	21, 32.8 (22.6 to 45.0)	3, 4.7 (1.6 to 12.9)	26, 40.6 (29.5 to 52.9)
(c) Pharmacists	9, 14.1 (7.6 to 24.6)	22, 34.4 (23.9 to 46.6)	2, 3.1 (0.9 to 10.7)	31, 48.4 (36.6 to 60.4)
(d) Auxiliary nurse	21, 32.8 (22.6 to 45.0)	6, 9.4 (4.4 to 19.0)	5, 7.8 (3.4 to 17.0)	32, 50.0 (38.1 to 61.9)
(e) Auxiliary nurse midwife	26, 40.6 (29.5 to 52.9)	3, 4.7 (1.6 to 12.9)	2, 3.1 (0.9 to 10.7)	33, 51.6 (39.6 to 63.4)
(f) Nurse	0, 0.0 (0.0 to 5.7)	2, 3.1 (0.9 to 10.7)	4, 6.3 (2.5 to 15.0)	58, 90.6 (81.0 to 95.6)
(g) Midwife	3, 4.7 (1.6 to 12.9)	2, 3.1 (0.9 to 10.7)	0, 0.0 (0.0 to 5.7)	59, 92.2 (83.0 to 96.6)
(h) Advanced associate clinician	15, 23.4 (14.7 to 35.1)	4, 6.3 (2.5 to 15.0)	1, 1.6 (0.3 to 8.3)	44, 68.8 (56.6 to 78.8)
(i) Non-specialist doctor	1, 1.6 (0.3 to 8.3)	4, 6.3 (2.5 to 15.0)	0, 0.0 (0.0 to 5.7)	59, 92.2 (83.0 to 96.6)
(j) Specialist doctor	0, 0.0 (0.0 to 5.7)	5, 7.8 (3.4 to 17.0)	1, 1.6 (0.3 to 8.3)	58, 90.6 (81.0 to 95.6)
Type of nutrition delivery platform				
Social protection programmes for vulnerable groups	11, 17.2 (9.9 to 28.2)	4, 6.3 (2.5 to 15.0)	13, 20.3 (12.3 to 31.7)	36, 56.3 (44.1 to 67.7)

For service delivery cadres, overlap was counted if that cadre was noted to deliver any FP service (information, education and counselling as well as any contraceptive method), plus any nutrition service (information, education and counselling or supplementation to pregnant or postpartum women and children, or food and cash assistance to any vulnerable group).

Overall percentages across all 64 countries are not weighted by the number of countries in each region.

CHW, community health worker; CI, confidence interval; FP, family planning.

## Discussion

These findings of the survey of health ministry officials in 64 member states of three WHO regions show differences in the reported FP and nutrition service provision. The characteristics of national FP and nutrition programmes varied by region and by country within each region. Countries in AFRO were most diverse in policies and programmes for FP and nutrition as well as outcomes related to the provision of these services. Respondents from over half of all countries indicated some level of integration of FP and nutrition services—with variations across regions in the exact nature of FP and nutrition services provided as part of integrated programmes. Recognition of potential value in integrating FP and nutrition services is notable, but not universal. In general, countries in EMRO lagged behind AFRO and SEARO on most indicators. Few EMRO countries reported currently integrated FP and nutrition programmes and interest in integration.

With over half of all countries reporting the existence of integrated FP counselling or method provision with nutritional services, and a varying level of the remaining countries reporting some option for integration, the interest and potential for FPN integration appeared substantial in the 64 countries surveyed. However, the interest in the integration of FP and nutrition and overlap of service delivery personnel was not universal. This may be due to a lack of recognition of biological links between improved FP and improved nutrition, perceived need for more evidence to justify integrating programmes and other structural or sociocultural barriers—as has been previously reported in qualitative investigations and reviews.^6 17 26^ While the survey did not explore qualitative reasons for non-integration of services, our data reinforce that approaches to integrating FP and nutrition services, if at all considered suitable, will need to be context-specific and take into account current government priorities and motivations. Furthermore, while it may be ideal to design programmes that may fully consider and address FP and nutrition needs during each life stage, given setting-specific constraints, the actual value and feasibility of aligning programmes may vary across countries depending on the health system structure and social, fiscal and political context.^17^

Nonetheless, and importantly, our data identify potentially easier ‘wins’ to promote integration in an opportunistic manner. We observed an overlap between staff most identified as key to delivering FP and nutrition services across countries: both FP and nutrition services were reported to be delivered by the same cadre of workers at the community and health facilities by a substantial proportion of countries, indicating strong structural possibilities to integrate. Identification and leveraging of these opportunities by health systems is key to a ‘diagonal approach’ to building services to improve women’s health effectively and efficiently.^3^

Our data also highlight the need for greater focus on more vulnerable groups in relation to FP and nutrition needs. In particular, FP and nutrition services were generally less focused on adolescents, despite increasing evidence of the importance of appropriate nutrition during this period for health and functional outcomes,^27 28^ as well as a persistent burden of adolescent pregnancy,^29^ which is understood to affect nutritional status as well as long-term health and socioeconomic outcomes.^7 10^ While targeting adolescent populations may be challenging, especially given the stigma surrounding FP use among unmarried girls,^16 30^ global tools for programme implementation exist to guide the delivery of packages that may appropriately serve adolescent FP and health needs, such as the Global Accelerated Action for the Health of Adolescent guidance, which includes nutrition and SRH in adolescents.^31^ Other existing social protection programmes also provide simple opportunities to co-deliver nutrition and FP interventions—with our data indicating that up to one-quarter of countries were only providing one or the other through such platforms. Recent reviews have indicated the benefits of social protection programmes for women’s empowerment, including through improved access to contraception and SRH knowledge and services, alongside improved children’s nutrition.^32^ This further supports our identification of such programmes as potentially key platforms to deliver integrated FP and nutrition services.

Modifying health systems requires resources, and research and advocacy will be important to support investment of resources to advance integration of FP and nutrition services effectively.^17^ However, as outlined above, there may be more opportunistic and low-resource approaches to provide integrated services based on the current structure of service delivery. Furthermore, given the mutually reinforcing benefits of FP and nutrition,^6 26^ improving access to nutrition and FP services as is will also be important to promote benefits. Our findings indicate that increasing efforts and investments in FP and nutrition programmes are required in EMRO, where programmes appeared to be generally less available in both domains. Furthermore, it would be valuable to identify and strengthen efforts to reach women who are most in need of nutritional and FP interventions but not visiting clinics, the principal platform for nutritional services—such as through community distribution of services.^15^ Furthermore, promoting task shifting of IEC in particular by non-specialist and specialist doctors to mid-level healthcare providers, especially auxiliary nurses and auxiliary nurse-midwives, may help to increase coverage.^33 34^

### Limitations

Great care was taken in identifying respondents most knowledgeable of national FP, nutrition and related SRH programmes and efforts were made by reminders (three maximum) to reach an adequate response rate. The use of an online platform allowed efficient data collection across multiple countries and regions, facilitated standardised administration of the questionnaire and enabled timely monitoring of responses. However, the electronic format also presented certain limitations, including potential exclusion of respondents with limited internet access or technical capacity, potential variability in interpretation of questions without in-person clarification, especially if feedback was sought from a secondary respondent and possible response bias due to self-administration. Furthermore, given that there was primarily one single respondent for each country, there may be potential for bias in responses based on their specific domain of expertise and work. While we did not find evidence of significant differences in responses in analyses stratified by domain of respondent, we cannot completely rule this out. Officials from most invited countries responded to our survey, and countries whose officials did not respond were similar in terms of key FP and nutrition indicators to those whose officials responded, although selection bias cannot fully be ruled out. Additionally, we did not conduct a formal assessment of questionnaire validity and reliability, focusing on face and content validity based on expert input and pretesting with region-specific respondents.

The survey comprised responses provided by the respondents rather than through observational study or collection of data directly from facilities. These responses do not reflect the fidelity to national guidelines, the quality or coverage of government services provided nor about the services provided in the private sector. Therefore, the possibility of response errors such as over-reporting or under-reporting cannot be ruled out. We also did not cover all elements in depth, including potential barriers to integrating FP and nutrition services.^17^ While this analysis provides a starting point for information regarding the availability of integrated services in theory, much further on-the-ground work would be important to better understand the reach of such services.

The respondents were health officials at the national level, and their responses may not be able to capture within-country variations. Also, given that there was only primarily one respondent per country, the reliability and validity of responses depend on the accuracy of information provided by them.

The response rate for EMRO was low (57%), which could raise the possibility of selectivity of countries responding to the survey in this region. The comparison across regions has the limitation of varying sample size by region and an overall small sample size for stratified analysis. Because of the small and non-comparable sample size of countries by region, we did not perform statistical tests for significance. We also did not weight overall estimates by the number of countries in each region, or account for any other country-specific characteristics such as population size. The survey was conducted in three WHO regions in LMICs in this phase. Countries in the Pan-American Health Organization and the Western Pacific region are not included in this survey.

In all, this study provides up-to-date data regarding the current landscape of FP and nutrition services across countries in three WHO regions, indicating some integration of services in these domains, recognition of nutrition and FP as being suitable for integration and overlap between cadres of workers delivering services. This survey suggests potential opportunities to explore strategies that may more strongly align FP and nutrition services to benefit women in LMICs.

## Supplementary material

10.1136/bmjgh-2025-020307 online supplemental file 1

10.1136/bmjgh-2025-020307 online supplemental file 2

## Data Availability

Data are available on reasonable request.
