# Supplementary material for: Integration of family planning and nutrition programmes in 64 WHO member states of Africa, Eastern Mediterranean and South-East Asia regions: findings from a survey of Ministry of Health officials
Source: BMJ Glob Health. 2026 Feb 17;10(Suppl 1):e020307. doi: 10.1136/bmjgh-2025-020307 (PMC12962060; doi:10.1136/bmjgh-2025-020307)

**Integration of Family Planning and Nutrition Programmes in 64 WHO member states of Africa, Eastern Mediterranean, and South-East Asia Regions: Findings from a survey of Ministry of Health Officials**

**Supplementary material**

**Supplementary Table A.1: Key indicators by WHO region and survey country**

|  | **Country** | **Population (000)** | **CPR (%)** | **mCPR (%)** | **Unmet Need (%)** | **ANC (%)** | **Anemia (women 15-49) (%)** | **Anemia (pregnant women) (%)** | **Mean Hemoglobin level (women 15-49) (g/L)** | **Mean**  **Hemoglobin level (pregnant women) (g/L)** |
| --- | --- | --- | --- | --- | --- | --- | --- | --- | --- | --- |
| **African Region (AFRO)** | |  |  |  |  |  |  |  |  |  |
| 1 | Burundi | 12,551 | 22.4 | 22.4 | 29.7 | 49.3 | 38.5 | 44.2 | 122 | 111 |
| 2 | Comoros | 822 | 14.2 | 14.2 | 31.6 | 48.9 | 33.8 | 38 | 124 | 114 |
| 3 | Eritrea | 3,620 | 7 | 7 | 27.4 | 57.4 | 37 | 40.8 | 123 | 113 |
| 4 | Ethiopia | 120,281 | 35.6 | 35.6 | 19.2 | 43 | 23.9 | 29 | 128 | 117 |
| 5 | Kenya | 53,006 | 60.1 | 60.1 | 14.1 | 66 | 28.7 | 40.3 | 127 | 113 |
| 6 | Madagascar | 28,916 | 42.2 | 42.2 | 14.6 | 59.9 | 37.8 | 39.3 | 123 | 114 |
| 7 | Malawi | 19,890 | 64.7 | 64.7 | 15.4 | 50.5 | 31.4 | 39.3 | 125 | 114 |
| 8 | Mauritius | 4,615 | 32 | 32 | 12.5 | 77.7 | 23.5 | 27.7 | 129 | 118 |
| 9 | Mozambique | 32,077 | 25.3 | 25.3 | 23.1 | 51 | 47.9 | 45.8 | 119 | 110 |
| 10 | Rwanda | 13,462 | 56.9 | 56.9 | 13.6 | 47.2 | 17.2 | 23.5 | 131 | 119 |
| 11 | South Sudan | 10,748 | 1.7 | 1.7 | 29.7 | 17 | 35.6 | 40 | 123 | 113 |
| 12 | Tanzania | 63,588 | 32 | 32 | 22.1 | 62.2 | 38.9 | 48.1 | 122 | 110 |
| 13 | Uganda | 45,854 | 42.7 | 42.7 | 15 | 56.7 | 32.8 | 38.9 | 125 | 114 |
| 14 | Zambia | 19,473 | 47.5 | 47.5 | 19.7 | 63.5 | 31.5 | 39.3 | 125 | 114 |
| 15 | Zimbabwe | 15,994 | 65.8 | 65.8 | 10.4 | 71.5 | 28.9 | 31.7 | 126 | 116 |
|  |  |  |  |  |  |  |  |  |  |  |
| 16 | Angola | 34,504 | 12.5 | 12.5 | 38 | 61.4 | 44.5 | 48.3 | 120 | 110 |
| 17 | Chad | 17,180 | 6.7 | 6.7 | 30.2 | 31 | 45.4 | 45.3 | 119 | 111 |
| 18 | Cameroon | 27,199 | 15 | 15 | 23 | 64.9 | 40.6 | 44.4 | 121 | 112 |
| 19 | Congo | 5,836 | 18.5 | 18.5 | 17.9 | 79 | 48.8 | 51 | 119 | 109 |
| 20 | Guinea Equatorial | 1,634 | 9.5 | 9.5 | 33.8 | 66.9 | 44.5 | 48.1 | 120 | 110 |
| 21 | Gabon | 2,341 | 19.4 | 19.4 | 26.5 | 77.6 | 52.4 | 52.5 | 117 | 108 |
| 22 | Dem Rep Congo | 95,894 | 17.6 | 17.6 | 28.7 | 56 | 42.4 | 46.5 | 111 | 121 |
|  |  |  |  |  |  |  |  |  |  |  |
| 23 | Botswana | 2,588 | 67.4 | 67.4 | 9.6 | 73.3 | 32.5 | 31.4 | 125 | 116 |
| 24 | Eswatini | 1,192 | 65.5 | 65.5 | 15.2 | 76.1 | 30.7 | 32.2 | 126 | 116 |
| 25 | Lesotho | 2,281 | 64.6 | 64.6 | 16 | 76.6 | 27.9 | 33.5 | 127 | 115 |
| 26 | Namibia | 2,530 | 55.3 | 55.3 | 17.5 | 62.5 | 25.2 | 29 | 128 | 117 |
| 27 | South Africa | 59,392 | 54 | 54 | 14.9 | 75.5 | 30.5 | 30.8 | 126 | 118 |
|  |  |  |  |  |  |  |  |  |  |  |
| 28 | Benin | 12,997 | 12 | 12 | 32.2 | 52.1 | 55.2 | 58.1 | 116 | 105 |
| 29 | Burkina Faso | 22,101 | 31.7 | 31.7 | 17 | 47.2 | 52.5 | 55.3 | 117 | 107 |
| 30 | Cabo Verde | 588 | 54.7 | 54.7 | 16.7 | 85.6 | 24.3 | 33.9 | 127 | 116 |
| 31 | Côte d'Ivoire | 27,478 | 21.8 | 21.8 | 20 | 51.3 | 50.9 | 54.4 | 117 | 107 |
| 32 | Ghana | 32,180 | 24.3 | 24.3 | 33.6 | 87.8 | 35.4 | 47.2 | 123 | 111 |
| 33 | Guiné-Bissau | 2,061 | 20.2 | 20.2 | 21.1 | 80.7 | 48.1 | 52.3 | 119 | 108 |
| 34 | Guinee | 13,532 | 10.3 | 10.3 | 22.1 | 58.3 | 48 | 53 | 118 | 108 |
| 35 | Liberia | 5,193 | 23.8 | 23.8 | 33.4 | 87.3 | 42.6 | 49.7 | 121 | 110 |
| 36 | Mauritania | 1,299 | 10 | 10 | 33.2 | 38.5 | 43.3 | 49.1 | 120 | 110 |
| 37 | Mali | 21,905 | 16.4 | 16.4 | 23.9 | 43.3 | 59 | 59 | 114 | 105 |
| 38 | Niger | 25,253 | 10 | 10 | 18 | 38.5 | 49.5 | 54.9 | 118 | 107 |
| 39 | Nigeria | 213,401 | 12.1 | 12.1 | 18.9 | 60.4 | 55.1 | 55 | 116 | 107 |
| 40 | Sierra Leone | 8,421 | 20.8 | 20.8 | 24.8 | 78.8 | 48.4 | 54.4 | 119 | 107 |
| 41 | Togo | 8,645 | 21.5 | 21.5 | 34 | 54.8 | 45.7 | 36.5 | 120 | 108 |
| 42 | Senegal | 16,877 | 25.4 | 25.4 | 21.7 | 55.5 | 52.7 | 56.1 | 117 | 107 |
| **South-East Asia Region (SEARO)** | |  |  |  |  |  |  |  |  |  |
| 43 | Bangladesh | 169,356 | 59.1 | 59.1 | 13.7 | 40.5 | 36.7 | 42.2 | 123 | 112 |
| 44 | Bhutan | 777 | 65.4 | 65.4 | 11.7 | 84.9 | 38.6 | 38.2 | 122 | 114 |
| 45 | India | 1,407,564 | 56.5 | 56.5 | 12.9 | 58.5 | 53 | 50.1 | 117 | 109 |
| 46 | Maldives | 521 | 14.7 | 14.7 | 31.4 | 81.6 | 52.2 | 49.3 | 118 | 110 |
| 47 | Nepal | 30,035 | 44.2 | 44.2 | 24.7 | 80.5 | 35.7 | 42.5 | 124 | 112 |
| 48 | Sri Lanka | 21,773 | 53.6 | 53.6 | 7.5 | 92.5 | 34.6 | 34.6 | 124 | 114 |
|  |  |  |  |  |  |  |  |  |  |  |
| 49 | Indonesia | 273,753 | 54.2 | 54.2 | 10.6 | 90.6 | 31.2 | 44.2 | 125 | 111 |
| 50 | Myanmar | 53,798 | 51.3 | 51.3 | 16.2 | 58.6 | 42.1 | 47.8 | 121 | 110 |
| 51 | Thailand | 71,601 | 71.3 | 71.3 | 8 | 90 | 24 | 32.2 | 128 | 116 |
| 52 | Timor-Leste | 1,321 | 23.7 | 23.7 | 25.3 | 76.7 | 29.9 | 38 | 125 | 114 |
| **Eastern Mediterranean Region (EMRO)** | |  |  |  |  |  |  |  |  |  |
| 53 | Djibouti | 1,106 | 18 | 18 | 26.7 | 25.7 | 32.3 | 37 | 125 | 114 |
| 54 | Somalia | 17,066 | 0.9 | 0.9 | 36.6 | 24.4 | 43.1 | 48.7 | 120 | 109 |
|  |  |  |  |  |  |  |  |  |  |  |
| 55 | Libya | 6,735 | 16.3 | 16.3 | 40.2 | . | 29.9 | 29.4 | 125 | 116 |
| 56 | Morocco | 37,077 | 59.1 | 59.1 | 11.3 | 60.9 | 29.9 | 32.6 | 126 | 116 |
|  |  |  |  |  |  |  |  |  |  |  |
| 57 | Gaza. Strip | . | . | . | . | . | . | . | . | . |
| 58 | Iraq | 43,534 | 36.1 | 36.1 | 14.3 | 67.9 | 28.6 | 30.9 | 124 | 115 |
| 59 | Jordan | 11,148 | 37.4 | 37.4 | 14.2 | 91.6 | 37.7 | 33.7 | 123 | 115 |
| 60 | Oman | 4,520 | 18.8 | 18.8 | 17.8 | 73 | 29.1 | 30.2 | 125 | 116 |
| 61 | Palestine | 5,133 | 42.8 | 42.8 | 12.9 | 94.8 | 31 | . | 126 | 115 |
| 62 | Yemen | 32,982 | 25.2 | 25.2 | 28.7 | 25.1 | 61.5 | 57.5 | 112 | 105 |
|  |  |  |  |  |  |  |  |  |  |  |
| 63 | Afghanistan | 40,099 | 17.4 | 17.4 | 24.5 | 33.4 | 42.6 | 36.5 | 120 | 115 |
| 64 | Pakistan | 231,402 | 23.4 | 23.4 | 17.3 | 52.4 | 41.3 | 44 | 120 | 111 |

Notes: CPR=Contraceptive prevalence of any method; mCPR=Contraceptive prevalence of modern methods; Unmet need=Unmet need for family planning; ANC=Antenatal care visits of at least four.

Data Sources: United Nations Department of Economic and Social Affairs, Population Division. 2022 World Population Prospects 2022: <https://population.un.org/wpp/Download/Standard/Population/>.

[United Nations United Nations Department of Economic and Social Affairs, Population Division. 2022. World Contraceptive Use 2022. POP/DB/CP/REV/2022/. https://www.un.org/development/desa/pd/data/world-contraceptive-use.](https://hu-my.sharepoint.com/personal/upartap_hsph_harvard_edu/Documents/Documents/Uttara%20Partap/6_FPN/4_Feedback/55_WHOSurvey/United%20Nations%20United%20Nations%20Department%20of%20Economic%20and%20Social%20Affairs,%20Population%20Division.%202022.%20World%20Contraceptive%20Use%202022.%20POP/DB/CP/REV/2022/.%20https:/www.un.org/development/desa/pd/data/world-contraceptive-use.)

https://www.who.int/data/gho/data/indicators/indicator-details/GHO/antenatal-care-coverage-at-least-four-visits.

| <https://www.who.int/data/gho/data/indicators/indicator-details/GHO/prevalence-of-anaemia-in-women-of-reproductive-age-(-).> | |
| --- | --- |
| [https://www.who.int/data/gho/data/indicators/indicator-details/GHO/prevalence-of-anaemia-in-pregnant-women-(-).](https://www.who.int/data/gho/data/indicators/indicator-details/GHO/prevalence-of-anaemia-in-pregnant-women-(-)) |  |
| [https://www.who.int/data/gho/data/indicators/indicator-details/GHO/mean-hemoglobin-level-of-women-of-reproductive-age-(aged-15-49-years).](https://www.who.int/data/gho/data/indicators/indicator-details/GHO/mean-hemoglobin-level-of-women-of-reproductive-age-(aged-15-49-years)) | |
| [https://www.who.int/data/gho/data/indicators/indicator-details/GHO/mean-hemoglobin-level-of-pregnant-women-(aged-15-49-years).](https://www.who.int/data/gho/data/indicators/indicator-details/GHO/mean-hemoglobin-level-of-pregnant-women-(aged-15-49-years)) | |

| **Supplementary Table A.2. Population-level differences in available key family planning and nutrition-related indicators between responding (N=64) and non-responding (N=15) countries.*** | | | | | | | | |
| --- | --- | --- | --- | --- | --- | --- | --- | --- |
|  | **mCPR (%)** | | **Unmet Need (%)** | | **Anemia among women 15-49 (%)** | | **Anemia among pregnant women 15-49 (%)** | |
|  | **Number of countries** | **Median (IQR)** | **Number of countries** | **Median (IQR)** | **Number of countries** | **Median (IQR)** | **Number of countries** | **Median (IQR)** |
| **Overall** |  |  |  |  |  |  |  |  |
| **Included** | 61 | 25.2 (17.4,53.6) | 63 | 19.7 (14.8, 28.1) | 63 | 37.8 (30.9, 46.8) | 62 | 42.4 (34.1, 49.3) |
| **Excluded** | 13 | 44.9 (34.3,48.4) | 10 | 16.0 (12.8, 25.3) | 14 | 29.6 26.1, 23.7) | 16 | 33.1 (28.6, 35.2) |
| **P** | **0.115** | | **0.275** | | **0.005** | | **<0.001** | |
| **AFRO** |  |  |  |  |  |  |  |  |
| **Included** | 41 | 23.8 (15.0, 47.5) | 42 | 21.4 (16.2, 29.5) | 42 | 39.8 (31.4, 48.1) | 42 | 44.9 (36.9, 52.0) |
| **Excluded** | 3 | 44.9 (29.7, 45.5) | 3 | 27.1 (20.6, 32.4) | 4 | 35.7 (30.1, 40.6 | 4 | 39.1 (33.2, 44.3) |
| **P** | **0.692** | | **0.601** | | **0.413** | | **0.311** | |
| **SEARO** |  |  |  |  |  |  |  |  |
| **Included** | 9 | 53.6 (44.2, 59.1) | 10 | 13.3 (10.9, 22.6) | 10 | 36.2 (32.1, 41.2) | 10 | 42.4 (33.2, 44.3) |
| **Excluded** | 1 | 68.8 | 1 | 6.6 | 1 | 27.5 | 1 | 29.7 |
| **P** | **0.223** | | **0.114** | | **0.206** | | **0.114** | |
| **EMRO** |  |  |  |  |  |  |  |  |
| **Included** | 11 | 23.4 (17.7, 36.8) | 11 | 17.8 (14.3, 27.7) | 11 | 31.7 (29.9, 42.0) | 10 | 35.1 (31.3, 42.3) |
| **Excluded** | 9 | 39.3 (34.3, 48.4) | 6 | 16.0 (13.2, 20.2) | 11 | 28.2 (26.6, 33.4) | 11 | 31.6 (28.5, 33.9) |
| **P** | **0.063** | | **0.269** | | **0.049** | | **0.029** | |
| P for differences between values examined using Mann-Whitney U Test.  Note: all available reported data from each country was used. Ns of respondents or non-respondents may be different for each indicator depending upon potentially missing data on country-level indicators. | | | | | | | | |

**Supplementary Table A.3: Number and percentage (%) of nutrition services provided by cadre of service provider and region**

| Type of service and cadre of health worker | AFRO N=42  n (%) | SEARO N=10  n (%) | EMRO N=12  n (%) | All three regions N=64  n (%) |
| --- | --- | --- | --- | --- |
| Information, education, and communication (IEC) only |  |  |  |  |
| a. Lay health worker (e.g., CHW) | 35 (83.3) | 7 (70.0) | 8 (66.7) | 50 (78.1) |
| b. Pharmacy worker | 11 (26.2) | 2 (20.0) | 1 (8.3) | 14 (21.9) |
| c. Pharmacist | 12 (28.6) | 4 (40.0) | 2 (16.7) | 18 (28.1) |
| d. Auxiliary nurse | 24 (57.1) | 5 (50.0) | 4 (33.3) | 33 (51.6) |
| e. Auxiliary nurse midwife | 21 (50.0) | 6 (60.0) | 4 (33.3) | 31 (48.4) |
| f. Nurse | 39 (92.9) | 10 (100.0) | 9 (75.0) | 58 (90.6) |
| g. Midwife | 40 (95.2) | 8 (80.0) | 9 (75.0) | 57 (89.1) |
| h. Advanced Associate Clinician | 30 (71.4) | 6 (60.0) | 5 (41.7) | 41 (64.1) |
| i. Non-specialist doctor | 37 (88.1) | 9 (90.0) | 10 (83.3) | 56 (87.5) |
| j. Specialist doctor | 38 (90.5) | 9 (90.0) | 10 (83.3) | 57 (89.1) |
| Nutrition counselling during pregnancy |  |  |  |  |
| a. Lay health worker (e.g., CHW) | 33 (78.6) | 8 (80.0) | 6 (50.0) | 47 (73.4) |
| b. Pharmacy worker | 7 (16.7) | 2 (20.0) | 1 (8.3) | 10 (15.6) |
| c. Pharmacist | 9 (21.4) | 4 (40.0) | 2 (16.7) | 15 (23.4) |
| d. Auxiliary nurse | 25 (59.5) | 5 (50.0) | 3 (25.0) | 33 (51.6) |
| e. Auxiliary nurse midwife | 22 (52.4) | 6 (60.0) | 4 (33.3) | 32 (50.0) |
| f. Nurse | 40 (95.2) | 10 (100.0) | 9 (75.0) | 59 (92.2) |
| g. Midwife | 40 (95.2) | 8 (80.0) | 9 (75.0) | 57 (89.1) |
| h. Advanced Associate Clinician | 32 (76.2) | 6 (60.0) | 2 (16.7) | 40 (62.5) |
| i. Non-specialist doctor | 38 (90.5) | 9 (90.0) | 8 (72.7) | 55 (87.3) |
| j. Specialist doctor | 37 (88.1) | 9 (90.0) | 10 (83.3) | 56 (87.5) |
| Daily Iron-Folic Acid (IFA) supplement to pregnant women |  |  |  |  |
| a. Lay health worker (e.g., CHW) | 25 (59.2) | 5 (50.0) | 0 (0.0) | 30 (49.2) |
| b. Pharmacy worker | 11 (26.2) | 3 (30.0) | 5 (41.7) | 19 (29.7) |
| c. Pharmacist | 15 (35.7) | 6 (60.0) | 6 (50.0) | 27 (42.2) |
| d. Auxiliary nurse | 22 (52.4) | 5 (50.0) | 3 (25.0) | 30 (46.9) |
| e. Auxiliary nurse midwife | 22 (52.4) | 6 (60.0) | 4 (33.3) | 32 (50.0) |
| f. Nurse | 37 (88.1) | 9 (90.0) | 7 (58.3) | 53 (82.8) |
| g. Midwife | 38 (90.5) | 8 (80.0) | 9 (75.0) | 55 (85.9) |
| h. Advanced Associate Clinician | 30 (71.4) | 6 (60.0) | 2 (16.7) | 38 (59.4) |
| i. Non-specialist doctor | 37 (88.1) | 8 (80.0) | 9 (75.0) | 54 (84.4) |
| j. Specialist doctor | 34 (81.0) | 9 (90.0) | 9 (75.0) | 52 (81.3) |
| Multiple-micronutrient (MMN) counselling during pregnancy |  |  |  |  |
| a. Lay health worker (e.g., CHW) | 14 (33.3) | 3 (30.0) | 2 (16.7) | 19 (30.0) |
| b. Pharmacy worker | 8 (19.1) | 3 (30.0) | 3 (25.0) | 14 (21.9) |
| c. Pharmacist | 11 (26.2) | 4 (40.0) | 5 (41.7) | 20 (31.3) |
| d. Auxiliary nurse | 15 (35.7) | 3 (30.0) | 2 (16.7) | 20 (31.3) |
| e. Auxiliary nurse midwife | 15 (35.7) | 4 (40.0) | 3 (25.0) | 22 (34.4) |
| f. Nurse | 29 (69.1) | 7 (70.0) | 4 33.3) | 40 (62.5) |
| g. Midwife | 30 (71.4) | 5 (50.0) | 5 (41.7) | 40 (62.5) |
| h. Advanced Associate Clinician | 25 (59.2) | 4 (40.0) | 1 (8.3) | 30 (46.9) |
| i. Non-specialist doctor | 29 (69.1) | 6 (60.0) | 7 (58.3) | 42 (65.6) |
| j. Specialist doctor | 30 (71.4) | 6 (60.0) | 7 (58.3) | 43 (67.2) |
| Breastfeeding counselling during pregnancy |  |  |  |  |
| a. Lay health worker (e.g., CHW) | 35 (83.3) | 7 (70.0) | 5 (41.7) | 47 (73.4) |
| b. Pharmacy worker | 10 (23.8) | 2 (20.0) | 3 (25.0) | 15 (23.4) |
| c. Pharmacist | 10 (23.8) | 4 (40.0) | 3 (25.0) | 17 (26.6) |
| d. Auxiliary nurse | 22 (52.4) | 5 (50.0) | 4 (33.3) | 31 (48.4) |
| e. Auxiliary nurse midwife | 19 (45.2) | 6 (60.0) | 5 (41.7) | 30 (46.9) |
| f. Nurse | 41 (97.6) | 10 (100.0) | 7 (58.3) | 58 (90.6) |
| g. Midwife | 38 (90.5) | 8 (80.0) | 10 (83.3) | 56 (87.5) |
| h. Advanced Associate Clinician | 31 (73.8) | 6 (60.0) | 3 (25.0) | 40 (62.5) |
| i. Non-specialist doctor | 35 (83.3) | 9 (90.0) | 9 (75.0) | 53 (82.8) |
| j. Specialist doctor | 34 (81.0) | 10 (100.0) | 10 (83.3) | 54 (84.4) |
| Breastfeeding counselling during the first two days after delivery |  |  |  |  |
| a. Lay health worker (e.g., CHW) | 29 (69.1) | 7 (70.0) | 5 (41.7) | 41 (64.1) |
| b. Pharmacy worker | 7 (16.7) | 2 (20.0) | 1 (8.3) | 10 (15.6) |
| c. Pharmacist | 7 (16.7) | 3 (30.0) | 1 (8.3) | 11 (17.2) |
| d. Auxiliary nurse | 19 (45.2) | 4 (40.0) | 3 (25.0) | 26 (40.6) |
| e. Auxiliary nurse midwife | 21 (50.0) | 6 (60.0) | 4 (33.3) | 31 (48.4) |
| f. Nurse | 37 (88.1) | 10 (100.0) | 6 (50.0) | 53 (82.1) |
| g. Midwife | 38 (90.5) | 8 (80.0) | 9 (75.0) | 55 (85.9) |
| h. Advanced Associate Clinician | 31 (73.8) | 6 (60.0) | 2 (16.7) | 39 (60.9) |
| i. Non-specialist doctor | 35 (83.3) | 9 (90.0) | 8 (66.7) | 52 (81.3) |
| j. Specialist doctor | 33 (78.6) | 10 (100.0) | 8 (66.7) | 51 (79.7) |
| Vitamin A supplementation among children 6-59 months |  |  |  |  |
| a. Lay health worker (e.g., CHW) | 28 (66.7) | 4 (40.0) | 3 (25.0) | 35 (54.7) |
| b. Pharmacy worker | 10 (23.8) | 2 (20.0) | 1 (8.3) | 13 (20.3) |
| c. Pharmacist | 12 (28.6) | 3 (30.0) | 1 (8.3) | 16 (25.0) |
| d. Auxiliary nurse | 20 (47.6) | 4 (40.0) | 4 (33.3) | 28 (43.8) |
| e. Auxiliary nurse midwife | 20 (47.6) | 5 (50.0) | 2 (16.7) | 27 (42.2) |
| f. Nurse | 37 (88.1) | 9 (90.0) | 7 (58.3) | 53 (82.8) |
| g. Midwife | 35 (83.3) | 8 (80.0) | 4 (33.3) | 47 (73.4) |
| h. Advanced Associate Clinician | 29 (69.1) | 5 (50.0) | 2 (16.7) | 36 (56.3) |
| i. Non-specialist doctor | 33 (78.6) | 7 (70.0) | 4 (33.3) | 44 (68.8) |
| j. Specialist doctor | 31 (73.8) | 8 (80.0) | 5 (41.7) | 44 (68.8) |
| Weekly Iron-Folic Acid (IFA) supplement to adolescents and reproductive age women |  |  |  |  |
| a. Lay health worker (e.g., CHW) | 14 (33.3) | 4 (40.0) | 2 (16.7) | 20 (31.3) |
| b. Pharmacy worker | 6 (14.3) | 3 (30.0) | 1 (8.3) | 10 (15.6) |
| c. Pharmacist | 9 (21.4) | 5 (50.0) | 1 (8.3) | 15 (23.4) |
| d. Auxiliary nurse | 14 (33.3) | 4 (40.0) | 1 (8.3) | 19 (29.7) |
| e. Auxiliary nurse midwife | 12 (28.6) | 4 (40.0) | 1 (8.3) | 17 (26.6) |
| f. Nurse | 25 (59.2) | 7 (70.0) | 0 (0.0) | 32 (50.0) |
| g. Midwife | 22 (52.4) | 7 (70.0) | 1 (8.3) | 30 (46.9) |
| h. Advanced Associate Clinician | 17 (40.5) | 5 (50.0) | 0 (0.0) | 22 (34.4) |
| i. Non-specialist doctor | 20 (47.6) | 6 (60.0) | 3 (25.0) | 29 (45.3) |
| j. Specialist doctor | 19 (45.2) | 6 (60.0) | 4 (33.3) | 29 (45.3) |
| Food and/or cash assistance to population groups |  |  |  |  |
| a. Lay health worker (e.g., CHW) | 14 (33.3) | 3 (30.0) | 1 (8.3) | 18 (28.1) |
| b. Pharmacy worker | 5 (11.9) | 0 (0.0) | 0 (0.0) | 5 (7.8) |
| c. Pharmacist | 5 (11.9) | 1 (10.0) | 0 (0.0) | 6 (9.4) |
| d. Auxiliary nurse | 9 (21.4) | 0 (0.0) | 0 (0.0) | 9 (14.1) |
| e. Auxiliary nurse midwife | 7 (16.7) | 1 (10.0) | 0 (0.0) | 8 (12.5) |
| f. Nurse | 14 (33.3) | 4 (40.0) | 0 (0.0) | 18 (28.1) |
| g. Midwife | 14 (33.3) | 3 (30.0) | 0 (0.0) | 17 (26.6) |
| h. Advanced Associate Clinician | 10 (23.8) | 1 (10.0) | 0 (0.0) | 11 (17.2) |
| i. Non-specialist doctor | 11 (26.2) | 2 (20.0) | 1 (8.3) | 14 (21.9) |
| j. Specialist doctor | 10 (23.8) | 2 (20.0) | 1 (8.3) | 13 (20.3) |

Note: AFRO=African Region; SEARO=South-east Asia Region; and EMRO=Eastern Mediterranean Region. FP=Family Planning

| **Supplementary Table A.4: Number and percentage (%) of countries indicating family planning currently integrated with nutrition services, by type of service and region and domain of the respondent.** | | | | | |
| --- | --- | --- | --- | --- | --- |
|  | **Family Planning** | **MNCH** | **SRH** | **Public Health or other** | **P for difference** |
| **FP and nutrition integration** | **n, % (95% CI)** | **n, % (95% CI)** | **n, % (95% CI)** | **n, % (95% CI)** |  |
| FP counselling currently integrated with nutrition services | 11,  57.9 (36.3, 76.9) | 10,  58.8 (36.0, 78.4) | 8,  72.7 (43.4, 90.3) | 11,  64.7 (41.3, 82.7) | 0.888 |
| FP method provision currently integrated with nutrition services | 8,  42.1 (23.1, 63.7) | 9,  52.9 (31.0, 73.8) | 6,  54.5 (28.0, 78.7) | 8,  47.1 (26.2, 69.0) | 0.892 |
| Specific nutritional service integrated with FP in 40 countries with FPN integrated programmes* | **N=11** | **N=10** | **N=8** | **N=11** |  |
| Nutritional information, education, and communication only | 11,  100.0 (74.1, 100.0) | 9,  90.0 (59.6, 98.2) | 8,  100.0 (67.6, 100.0) | 11,  100.0 (74.1, 100.0) | 0.450 |
| Vitamin A supplementation among children 6-59 months | 7,  63.6 (35.4, 84.8) | 5,  50.0 (23.7, 76.3) | 6,  75.0 (40.9, 92.9) | 7,  63.6 (35.4, 84.8) | 0.766 |
| Weekly Iron Folic Acid (IFA) supplement for women aged 15-49 years | 6,  54.5 (28.0, 78.7) | 5,  50.0 (23.7, 76.3) | 4,  50.0 (21.5, 78.5) | 5,  45.5 (21.3, 72.0) | 1.000 |
| Daily Iron Folic Acid (IFA) supplement for pregnant women | 8,  72.7 (43.4, 90.3) | 7,  70.0 (39.7, 89.2) | 7,  87.5 (52.9, 97.8) | 10,  90.9 (62.3, 98.4) | 0.621 |
| Multiple micronutrient (MMN) supplement for pregnant women | 7,  63.6 (35.4, 84.8) | 7,  70.0 (39.7, 89.2) | 5,  62.5 (30.6, 86.3) | 5,  45.5 (21.3, 72.0) | 0.705 |
| Food and/or cash assistance to population groups | 7,  63.6 (35.4, 84.8) | 6,  60.0 (31.3, 83.2) | 6,  75.0 (40.9, 92.9) | 2,  18.2 (5.2, 47.7) | 0.059 |
| Nutrition counselling during pregnancy | 11,  100.0 (74.1, 100.0) | 9,  90.0 (59.6, 98.2) | 8,  100.0 (67.6, 100.0) | 11,  100.0 (74.1, 100.0) | 0.450 |
| Breastfeeding counselling during pregnancy | 10,  90.9 (62.3, 98.4) | 9,  90.0 (59.6, 98.2) | 8,  100.0 (67.6, 100.0) | 11,  100.0 (74.1, 100.0) | 0.845 |
| Breastfeeding counselling during the first two days after delivery | 11,  100.0 (74.1, 100.0) | 10,  100.0 (72.2, 100.0) | 8,  100.0 (67.6, 100.0) | 11,  100.0 (74.1, 100.0) | NA |
| *Countries that reported integration of FP counselling or method provision with nutrition are the sample (denominator) for the analysis. | | | | | |
| Note: AFRO=African Region; SEARO=South-east Asia Region; and EMRO=Eastern Mediterranean Region. FP=Family Planning. 95% CI: 95%confidence interval.  P values calculated using Pearson’s Chi squared tests, or Fisher’s exact tests if any cell count was <5. | | | | | |

| **Supplementary Table A.5: Number and percentage (%) of countries with currently no integrated family planning and nutrition programmes indicating positively the specific nutritional service for integration with family planning services, stratified by the domain of the respondent.** | | | | | |
| --- | --- | --- | --- | --- | --- |
|  | **Family Planning**  **(N=8)** | **MNCH**  **(N=7)** | **SRH**  **(N=3)** | **Public Health or other (N=6)** | **P for difference** |
| **FP and nutrition integration** | **n, % (95% CI)** | **n, % (95% CI)** | **n, % (95% CI)** | **n, % (95% CI)** |  |
| Specific nutritional service for integration with FP in 24 countries with no FPN integrated programmes* |  | | | | |
| Nutritional information, education, and communication only | 4,  50.0 (21.5, 78.5) | 1,  14.3 (2.6, 51.3) | 1,  33.3 (6.1, 79.2) | 0,  0.0 (0.0, 39.0) | 0.174 |
| Vitamin A supplementation among children 6-59 months | 3,  37.5 (13.7, 69.4) | 2,  28.6 (8.2, 64.1) | 2,  66.7 (20.8, 93.9) | 2,  33.3 (9.7, 70.0) | 0.783 |
| Weekly Iron Folic Acid (IFA) supplement for women aged 15-49 years | 4,  50.0 (21.5, 78.5) | 2,  28.6 (8.2, 64.1) | 2,  66.7 (20.8, 93.9) | 3,  50.0 (18.8, 81.2) | 0.759 |
| Daily Iron Folic Acid (IFA) supplement for pregnant women | 3,  37.5 (13.7, 69.4) | 1,  14.3 (2.6, 51.3) | 2,  66.7 (20.8, 93.9) | 2,  33.3 (9.7, 70.0) | 0.481 |
| Multiple micronutrient (MMN) supplement for pregnant women | 3,  37.5 (13.7, 69.4) | 1,  14.3 (2.6, 51.3) | 2,  66.7 (20.8, 93.9) | 3,  50.0 (18.8, 81.2) | 0.394 |
| Food and/or cash assistance to population groups | 5,  62.5 (30.6, 86.3) | 2,  28.6 (8.2, 64.1) | 2,  66.7 (20.8, 93.9) | 4,  66.7 (30.0, 90.3) | 0.539 |
| Nutrition counselling during pregnancy | 5,  62.5 (30.6, 86.3) | 1,  14.3 (2.6, 51.3) | 2,  66.7 (20.8, 93.9) | 1,  16.7 (3.0, 56.4) | 0.122 |
| Breastfeeding counselling during pregnancy | 4,  50.0 (21.5, 78.5) | 1,  14.3 (2.6, 51.3) | 2,  66.7 (20.8, 93.9) | 2,  33.3 (9.7, 70.0) | 0.376 |
| Breastfeeding counselling during the first two days after delivery | 4,  50.0 (21.5, 78.5) | 1,  14.3 (2.6, 51.3) | 2,  66.7 (20.8, 93.9) | 2,  33.3 (9.7, 70.0) | 0.376 |
| *Countries that reported **no** integration of FP counselling **or** method provision with nutrition are the sample (denominator) for the analysis. | | | | | |
| Note: AFRO=African Region; SEARO=South-east Asia Region; and EMRO=Eastern Mediterranean Region. FP=Family Planning. 95% CI: 95%confidence interval. | | | | | |

**Supplementary Figure A.1: Percentage of 64 countries with reported provision of information, education and communication (IEC) on family planning and nutrition by cadre of service provider**


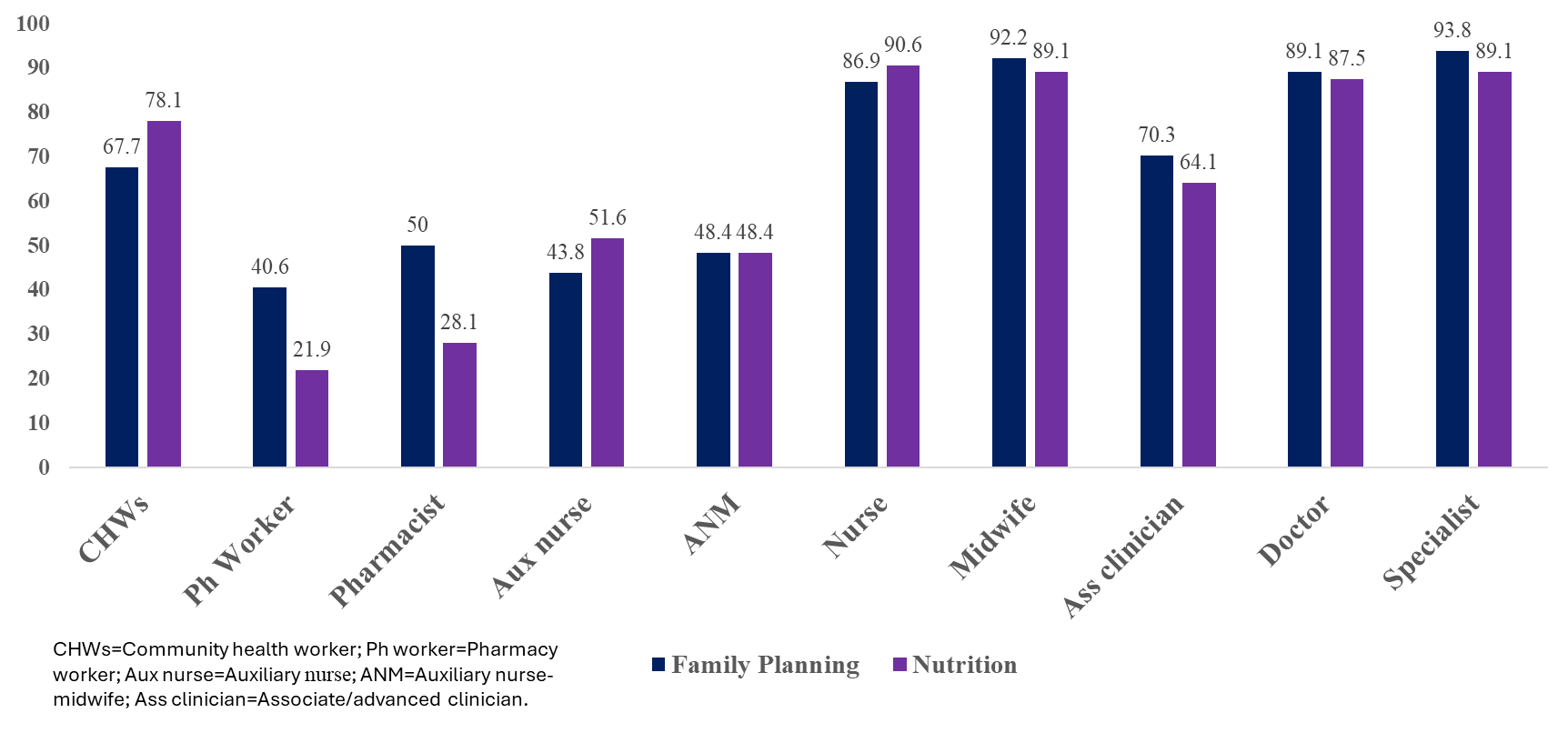

Supplement: online supplemental file 1 [file bmjgh-10-Suppl_1-s001.docx]
