# Supplementary material for: Integration of family planning and nutrition programmes in 64 WHO member states of Africa, Eastern Mediterranean and South-East Asia regions: findings from a survey of Ministry of Health officials
Source: BMJ Glob Health. 2026 Feb 17;10(Suppl 1):e020307. doi: 10.1136/bmjgh-2025-020307 (PMC12962060; doi:10.1136/bmjgh-2025-020307)
Supplement: online supplemental file 2 [file bmjgh-10-Suppl_1-s002.docx]

### BMJ Global Health Author Reflexivity Statement

Adapted from Morton, B., Vercueil, A., Masekela, R., Heinz, E., Reimer, L., Saleh, S., Kalinga, C., Seekles, M., Biccard, B., Chakaya, J., Abimbola, S., Obasi, A. and Oriyo, N. (2022), Consensus statement on measures to promote equitable authorship in the publication of research from international partnerships. Anaesthesia, 77: 264-276. <https://doi.org/10.1111/anae.15597>

| **Study conceptualisation** | |
| --- | --- |
| 1. How does this study address local research and policy priorities? | This study aims to understand the extent to which family planning and nutrition services may be integrated to provide the most efficient and impactful care to women of reproductive age, across three key regions where family planning access and nutritional status among women generally remains poor (WHO-defined South East Asia, Eastern Mediterranean, and Africa regions). This information is key to identifying where possible gaps are to improve care for (and the health status of) women in these countries. |
| 1. How were local researchers involved in study design? | Experts from each of the regions of interest were involved in the design and testing of the survey that was used to ensure sufficient relevance. |
| **Research management** | |
| 1. How has funding been used to support the local research team(s)? | The data collection teams at each WHO regional and country office were supported for their time in development, testing, inviting respondents and distributing the electronic questionnaire to respondents, as well for data analysis and interpretation. |
| **Data acquisition and analysis** | |
| 1. How are research staff who conducted data collection acknowledged? | Data were collected via an online survey, and data collection was facilitated by teams at the WHO regional and country offices. Named co-authors in this manuscript led the data collection process and are based at the regional offices (LO, AA, KG, MTU, and AJ). For country office staff who did not meet the ICMJE criteria for authorship but were fundamental to the conduct of the study, we have therefore acknowledged their contribution in the acknowledgements section. |
| 1. How have members of the research partnership been provided with access to study data? | The study data are available to all members of this research partnership and are held and transferred securely and digitally. |
| 1. How were data used to develop analytical skills within the partnership? | All data analysis was undertaken by co-authors who are based at the partnering WHO regional and central offices. The data analysts who undertook this particular analysis had existing skills to undertake it. However, the dataset is currently being used for additional analyses as part of this partnership, under approaches which more explicitly seek to train and encourage skills development of colleagues in analysing the rich data available. |
| **Data interpretation** | |
| 1. How have research partners collaborated in interpreting study data? | All research partners reviewed the available data and provided input on analyses and the interpretation of the results. Research partners were engaged from the study development phase to the manuscript writing phase and through to submission and revision. |
| **Drafting and revising for intellectual content** | |
| 1. How were research partners supported to develop writing skills? | The writing of the manuscript was primarily done by IS, with support from MA, UP and WF. Research partners read manuscript drafts and were requested for critical input, providing an opportunity for editing the manuscript. |
| 1. How will research products be shared to address local needs? | Research products from this study will be fed back to each participating member state to help inform further action. The WHO has strong links with ministries of health in each country of interest, and feedback mechanisms exist to share back findings. Country-specific ministries of health are the best placed to critically assess and use the information from this study. |
| **Authorship** | |
| 1. How is the leadership, contribution and ownership of this work by LMIC researchers recognised within the authorship? | All co-authors in this manuscript are originally from LMICs. Many co-authors are still based in institutions at LMICs. |
| 1. How have early career researchers across the partnership been included within the authorship team? | Early- to mid-career researchers are included in the manuscript, including AAB and UP. |
| 1. How has gender balance been addressed within the authorship? | Five out of 11 coauthors in this manuscript are female. |
| **Training** | |
| 1. How has the project contributed to training of LMIC researchers? | As highlighted earlier, early- to mid-career researchers from LMICs have been included in this project, and have received hands-on opportunities for training and skills development through this project. WHO Regional staff were trained in development of survey questionnaire, data collection and follow up. Statisticians (AAB and NH) were trained in tabulations plans and statistical analysis. |
| **Infrastructure** | |
| 1. How has the project contributed to improvements in local infrastructure? | Because this project was an online survey of ministry officials with one target official in each country, it has not directly contributed to improvements in local infrastructure on the ground in the countries of interest. |
| **Governance** | |
| 1. What safeguarding procedures were used to protect local study participants and researchers? | We sought ethical review of the study prior to undertaking it and were provided with an ethical waiver from the review board of the World Health Organization. We did not collect any sensitive information from researchers. All data are held and analysed securely. |
